# Brief Counseling on Secondhand Smoke Exposure in Pregnant Women in Argentina and Uruguay

**DOI:** 10.3390/ijerph14010028

**Published:** 2016-12-29

**Authors:** Alicia Alemán, Paola Morello, Mercedes Colomar, Laura Llambi, Mabel Berrueta, Luz Gibbons, Pierre Buekens, Fernando Althabe

**Affiliations:** 1 Montevideo Clinical and Epidemiological Research Unit, Montevideo 11600, Uruguay; mcolomar@unicem-web.org; 2 Departamento de Investigación en Salud de la Madre y el Niño, Instituto de Efectividad Clínica y Sanitaria (IECS), Buenos Aires C1414CPV, Argentina; pmorello@msal.gov.ar (P.M.); mberrueta@iecs.org.ar (M.B.); lgibbons@iecs.org.ar (L.G.); falthabe@iecs.org.ar (F.A.); 3 Hospital de Clínicas, Facultad de Medicina, Universidad de la Republica, Montevideo 11600, Uruguay; llambil@hc.edu.uy; 4 School of Public Health and Tropical Medicine, Tulane University, New Orleans, LA 70112, USA; pbuekens@tulane.edu

**Keywords:** brief counseling, secondhand smoke exposure, pregnancy, antenatal care

## Abstract

Argentina and Uruguay have a high prevalence of smoking during pregnancy, as well as of secondhand smoke (SHS) exposure. In this secondary analysis of a trial to implement brief smoking cessation counseling during antenatal care in Argentina and Uruguay, we aim to evaluate the effects of the intervention on the rates of self-reported SHS exposure at home and at work, and on attitudes recalled by non-smoker women enrolled in the intervention group compared with the control group. We randomly assigned (1:1) 20 antenatal care clusters in Argentina and Uruguay to receive a multifaceted intervention to implement brief smoking cessation counseling, which also included questions and counseling regarding SHS exposure, or to receive the standard of care. There was not a statistically significant difference between groups of the intervention’s effect (reduction of exposure to SHS) on any of the three exposure outcome measures (exposure at home, work or other indoor areas) or on the attitudes of women regarding exposure (avoiding breathing SHS and having rooms where smoking is forbidden). This analysis shows that we should not expect reductions in SHS exposure with this modest intervention alone. To achieve such reductions, strategies engaging partners and other household members may be more effective.

## 1. Introduction

Secondhand smoke (SHS) exposure during pregnancy is associated with many adverse outcomes for both the mother and the newborn [1,2]. Pregnant women exposed to SHS are 16% more likely to have a low-birth-weight baby [3,4], 20% more likely to have a preterm birth [5] and 23% more likely to suffer a stillbirth [5,6]. Offspring also experience adverse health effects when mothers are exposed to SHS during pregnancy [7,8,9,10]. Exposure to SHS is high in southern Latin American countries [11]. A recent study conducted in Argentina and Uruguay reported that in both countries, 35.9% of pregnant women are exposed to SHS at home, work or in public places [12].

World Health Organization (WHO) guidelines released in 2013 strongly recommend that antenatal care (ANC) providers, at minimum, screen all pregnant women for both tobacco use and SHS exposure, provide brief tobacco cessation counseling, provide education regarding the harms of SHS and offer strategies to reduce SHS exposure at home [13]. To increase adherence to these recommendations in Argentina and Uruguay, we designed and evaluated a multifaceted strategy to promote screening for tobacco exposure, counseling for tobacco cessation and reduction of SHS exposure for pregnant women in ANC clinics in Buenos Aires and Montevideo [14]. This intervention showed a statistically significant absolute increase in the rates of screening for SHS exposure of 24% within the home and of 33% at work, compared to the control group. The intervention also reported a 30% statistically significant increase in education about the impact of SHS on maternal and child health.

Given that the evaluation of the intervention’s effect on SHS exposure was not among the trial objectives, it was not analyzed and reported in the main trial paper. However, it is important to assess whether the intervention’s positive effect on the rates of SHS exposure screening and education resulted in a subsequent reduction in the exposure to SHS reported by the women.

In this secondary analysis, we aim to evaluate the effects of the multifaceted intervention on the rates of SHS exposure at home and at work recalled by women enrolled in the intervention group compared to the control group. Additionally, we will evaluate the effect on the women’s attitudes towards SHS.

## 2. Materials and Methods

### 2.1. Study Design and Participants

This is a secondary analysis of the main paper called “A multifaceted strategy to implement brief smoking cessation counseling during antenatal care in Argentina and Uruguay: a cluster randomized trial” [14]. The primary study was a two-arm parallel cluster randomized trial with baseline and follow-up cross-sectional measurements. In summary, the intervention under evaluation was a brief counseling session based on the “Five As” strategy for tobacco cessation (Ask, Advise, Assess, Assist and Arrange), as well as a brief session related to SHS. In the intervention clusters, motivated midwives and physicians were identified as facilitators who would receive training to implement the intervention in their clinics. They participated in a two-day training workshop (planned and developed by a “Five As” expert training team) that included information on prevalence of tobacco use, health consequences of smoking, SHS exposure during pregnancy and the “Five As” intervention (including screening and advice related to SHS), as well as instruction in motivational interviewing and practice with role play exercises. On the last day of the workshop, each facilitator developed an implementation strategy to ensure that all women attending intervention clinics for ANC will receive the “Five As” counseling at every visit. The facilitator team, with the support of the “Five As” expert team, was responsible for training the rest of the providers in the intervention clinics. Posters showing the risks of smoking and exposure to SHS were exhibited in the intervention clinics and brochures with similar messages were given to women during ANC visits in the same clinics. Regarding SHS advice, pregnant women were counseled during antenatal care visits about the harms of SHS to themselves or to their unborn infant. Within the “Advise” component, women were encouraged to make their homes smoke-free by telling them it was one of the most important things they could do for the health of their families. Providers stressed their advice by explaining that there is no safe level of exposure to SHS, and that it cannot be controlled with ventilation, air conditioning, or by separating smokers from non-smokers. The control group did not receive any particular training or intervention, but was not limited from implementing other strategies for smoking cessation.

Data were collected during a baseline period of 6-months before randomization and during the last six months of the 18-month intervention (follow up) that lasted from 3 October 2011 to 29 November 2013. Approval for the study was obtained from the ethics review boards of all participating local and partner institutions. Study methods and outcomes are reported elsewhere [14,15].

All subjects gave their informed consent for inclusion before they participated in the study. The study was conducted in accordance with the Declaration of Helsinki, and the protocol was approved by the Ethics Committee of Tulane University Biomedical IRB, USA (Project identification code 141842-4), CEMIC Comite de Etica en Investigación Centro de Educación médica e Investigaciones Clínicas “Norberto Quirno”, Argentina (Project identification code 524) and Ethics Committee of the School of Medicine, Universidad de la República, Uruguay. (Project File number N° 071140-000347-10).

### 2.2. Exposure and Outcomes Considered for This Analysis

This analysis will compare the recall of SHS exposure and attitudes towards SHS in women receiving ANC at clinics assigned to the intervention group compared to those women receiving care in clinics in the control group. Women were classified per their smoking status as non-smokers, early quitters, late quitters or continuing smokers. Non-smokers were those who reported they had never smoked, had tried cigarettes but did not smoke regularly, or quit smoking before they found out that they were pregnant. Early quitters were women who reported quitting as soon as they found out that they were pregnant, did not smoke until delivery, and had saliva cotinine levels ≤10 ng/mL. Late quitters were women who were smokers, but quit later during pregnancy and remained abstinent until delivery, and had saliva cotinine levels ≤10 ng/mL. This analysis only includes non-smokers and smokers women who quit during pregnancy, either early or late quitters.

SHS exposure at home was considered positive if women reported that smoking was allowed in her home always, on certain occasions or in certain rooms and if her partner or another household member who lived with her smoked.

SHS exposure at work was considered positive if smoking was allowed at the woman´s workplace and if anyone at work smoked indoors in the last 30 days. SHS exposure indoors was considered positive if the woman reported always or sometimes being around smokers indoors (home, work or public places).

The attitudes towards SHS exposure were measured by asking women if they tried to avoid breathing smoke exhaled by people smoking near them and if there were rooms in their home where smoking was forbidden.

### 2.3. Statistical Analysis

We tested the hypothesis that the intervention decreased the frequency of women non-smokers and quitters who recalled exposure to SHS. The analysis was intention-to treat. The woman was considered the unit of analysis because we wanted to study the intervention effect at the individual level. For that analysis, we fit a model in which the variables included were the intervention, the time (baseline and follow-up) and the “intervention by time” interaction. To test the effect of the intervention, we focused on the significance of the interaction. The effect of the intervention on SHS exposure was assessed by calculating the relative odds ratio (ROR), the ratio of the odds ratio for the intervention group to the odds ratio for the control group (a ROR estimated as statistically different from one implies a significant intervention effect). To estimate our model, we used a Generalized Estimation Equation (GEE) to adjust for the correlation of women who belong to the same cluster. We considered a binomial distribution and the logit of the link function and we assumed an exchangeable working correlation structure. For each outcome variable, we reported the proportion of the presence of the outcome at baseline and in the follow-up period and the odds ratio (OR) comparing the two periods for each intervention and control group. Finally, the ROR and the corresponding *p*-value was reported. Analyses were conducted using SAS version 9.3 (SAS Institute Inc., Cary, NC, USA).

The effect of the intervention on women´s attitudes towards SHS exposure was analyzed using the same approach. The hypothesis was whether the intervention increased women’s attitudes towards avoiding SHS. The sample size was not powerful enough to detect if the effect was statistically different between Argentina and Uruguay. However, we present the results by country to allow for an evaluation of clinical heterogeneity.

## 3. Results

**Figure 1:**
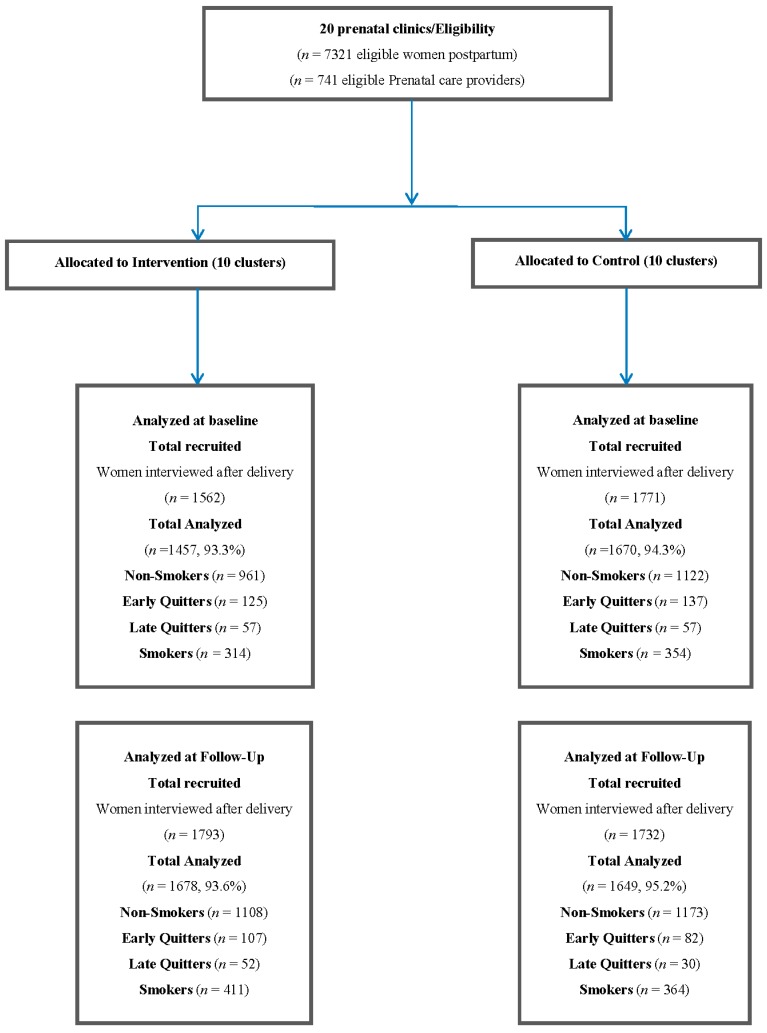
Trial diagram.

Twenty clusters completed the trial: 10 were randomized to the intervention group and 10 to the control group. The population of interest for this analysis was women who had data on smoking status, exposure to SHS, and were classified as non-smokers or quitters. Data from the baseline period were analyzed in 1143 women from the intervention group and in 1316 women from the control group. Follow-up period data were analyzed in 1267 and 1285 women in the intervention and control groups, respectively. Missing data were less than 6.7% overall (Figure 1).

The characteristics of the clusters and ANC providers were comparable between the intervention and control groups [14]. The groups were also similar with respect to maternal and newborn characteristics, the number of ANC visits, the rates of women recalling the “Five As” during ANC visits, and smoking status and SHS exposure during pregnancy. The proportion of women living in smoke-free homes was 45.9% vs. 42.1% in the intervention and control groups, respectively, and women having a partner/household member who smoked was 6.7% vs. 7.5% in the intervention and control groups, respectively.

Table 1 summarizes the effect of the intervention on SHS exposure in women non-smokers and quitters. Recalling SHS exposure at home or at work or reporting being always or sometimes around smokers at home, work or in public places was not significantly reduced between baseline and follow-up, either in the intervention or control groups. It is worth mentioning that, although non-statistically significant, SHS exposure at work showed a substantial decrease from 20.3% at baseline to 9.8% at follow-up in the intervention group, while there was a slight increase from 11.3% to 13.9% in the control group. The large imbalance of SHS exposure at work between groups at baseline was likely a result of chance and caused by high rates reported in three of the 10 clusters (the median of cluster rates was 9.1%). The relative difference between baseline and follow-up changes in the intervention and control groups (the intervention effect) was not statistically significant for any of the three outcome measures. Similarly, no differences were observed when women were stratified into non-smokers and quitters.

Similarly, no statistically significant differences were found for the intervention’s effect by country. However, it is worth mentioning that, while the recall of SHS exposure tended to decrease between baseline and follow-up in the intervention and control groups in Argentina, an opposite increasing trend was observed in Uruguay (Table 2).

The intervention was not effective in changing women’s attitudes regarding SHS exposure. There were no statistically significant differences between the intervention and the control clusters regarding the proportion of women who reported that they always or sometimes avoided breathing smoke exhaled by people smoking near them before and after the intervention. There were also no statistically significant differences between groups regarding the proportion of women who reported having rooms in their homes where smoking was forbidden (Table 3). Similarly, no differences were found when the analysis was stratified by non-smokers or quitters, or by country (data not shown).

## 4. Discussion

This secondary analysis assessed whether a multifaceted intervention shown to be effective in increasing SHS exposure screening and counseling during ANC, provided to women non-smokers and quitters, led to reductions in the reported SHS exposure and changed attitudes towards avoiding SHS. Despite the previously observed effects on providers’ behaviors, the intervention did not significantly modify the rate of women recalling SHS exposure at home, at work, or reporting being always or sometimes around smokers at home, at work or in public places. The intervention also did not affect women’s attitudes towards avoiding SHS. The effects were similar in both countries. However, while there was a decrease in SHS exposure between baseline and follow-up in Argentina, SHS exposure increased slightly in the same period in Uruguay

As we mentioned in the introduction, the intervention led to an increase in the rates of screening and counseling on SHS exposure at home and at work compared to the control group. However, at follow-up, the median rate of women in the intervention group who recalled advice on SHS exposure at home and at work was 67.4% and 48.3%, respectively. The median rate of women in the intervention group who recalled advice on SHS consequences on maternal and child health was 56.5% and 57.8%, respectively (data not published). Thus, the limited coverage of the different types of advice could have not been enough to produce a change in exposure behaviors in the overall group of women.

Another likely explanation for the lack of effect on the exposure is that the multifaceted intervention did not include specific components or tools to deal with SH, other than advice. Interventions reducing SHS exposure at home may require the implementation of smoke-free policies and components to support smoking cessation by household members. In a cluster randomized trial, Yang et al. showed that an intensive multi-component intervention consisting of three hospital-based group educational activities, clinician advice at prenatal visits, and brief monthly follow up telephone calls, plus educational materials and resources, was effective in reducing SHS exposure in women with a husband who smokes [16]. Similarly, the systematic review by Tong et al. evaluated studies that included not only brief counseling to non-smoking pregnant women, but also other components such as encouraging smoke-free rules, negotiation skills, or nicotine replacement therapy (NRT) to partners [17]. All of these studies reported that women in the intervention groups were more likely to have less exposure to SHS than women in the control groups. A recently published systematic review of five studies assessing multifaceted interventions for reducing SHS exposure in China [18] also showed positive results including increased quitting attempts by husbands and a higher proportion of women asking people to avoid smoking in their presence, compared to control groups [19].

Of the multiple barriers identified for establishing and maintaining smoke-free homes, our intervention only addressed one: the awareness and knowledge of the risks related to exposure to SHS [20]. Thirty-four percent of the women reported that household members smoked, but it was not a component of our intervention to provide cessation support to partners or household members [15].

The intervention did not include specific components targeting SHS at work either. Both countries already have bans for smoking in the work place [21,22]. Although not statistically significant, we did observe a 67% relative reduction between the intervention and control groups in SHS exposure at work. This reduction can also be explained by the imbalance in the rates of SHS at work observed at baseline between the intervention and control groups (20.3% and 11.3%, respectively) and a subsequent regression towards the mean [23]. Additionally, because this outcome was only assessed in women who worked outside the home, the small sample size makes chance a likely explanation.

Regarding attitudes towards exposure to SHS, our intervention did not show any effect. However, given that baseline rates were over 85% in both groups, there was minimal room for improvement.

To our knowledge, there are only two implementation trials that evaluated comparable smoking cessation interventions during pregnancy [18,24]. However, none reported effects on SHS exposure.

Finally, the slight increase in SHS exposure between baseline and follow-up in Uruguay, although not statistically significant, is consistent with the previously observed similar trend in smoking during pregnancy reported in the trial’s main paper [14]. This may be a matter of concern for public health in Uruguay.

## 5. Conclusions

### Strengths and Limitations

The study had several strengths already described in the main trial report [14]. Briefly, we used a rigorous experimental design; the groups were comparable and the outcome data collection was well separated from the intervention teams to prevent observer bias of the women’s outcomes. The study had a few limitations, however. Interviewing women during the postpartum stay and not during pregnancy could have affected recall of the ANC process, increasing outcome misclassification. Furthermore, this secondary analysis has limitations, namely that SHS exposure was not among the study outcomes. SHS exposure was measured by women’s recall with no biochemical validation, which has been shown to underestimate SHS exposure in the general population [25,26,27]. Additionally, the sample size was not originally calculated to evaluate SHS exposure as an outcome in non-smoking women. Thus, the potential underestimation of the exposure plus the limited sample size might have limited the power to detect an actual reduction in the exposure associated with the intervention. On the other hand, because women self-reported SHS exposure, we cannot rule out the possibility of a social desirability bias in the responses of women interviewed in the intervention group. However, this is unlikely given that the outcome was assessed at maternity hospitals that are largely independent of the ANC clinics.

As we have shown in the main trial paper, similar interventions may result in an increase in screening for SHS exposure and counseling about its consequences for pregnant women and, therefore, merit implementation at prenatal care visits [14]. However, this analysis shows that we should not expect large reductions in SHS exposure, although we cannot rule out some level of potentially important reductions in SHS exposure that were undetectable due to study limitations. To achieve large reductions, this intervention could be accompanied by more powerful strategies that remain unproven in our settings. Thus, future, more powerful studies are needed to test innovative interventions to reduce SHS exposure during pregnancy. Learning how to promote smoke-free homes is particularly important in countries where the prevalence of smoking is very high among men and lower among women, such as Argentina and Uruguay [12]. Finally, the trends of SHS exposure during pregnancy, mainly among low-income groups, should be closely monitored in these countries to assess whether our observations are isolated or are part of a more generalized problem [14].

## Figures and Tables

**Table 1:** Effect of intervention on secondhand smoke exposure on non-smokers and quitters globally.

	Intervention Group (10 Clusters)				Control Group (10 Clusters)				Intervention Effect	
	Baseline Rate (*n* = 1143)	Follow-Up Period Rate (*n* = 1267)	OR ^†^	*p*-Value *	Baseline Rate (*n* = 1316)	Follow-Up Period Rate (*n* = 1285)	OR ^†^	*p*-Value *	ROR ^‡^ (95% CI)	*p*-Value ^§^
SHS at home	23.1	19.6	0.83 (0.63–1.09)	0.1809	23.1	18.0	0.73 (0.55–0.97)	0.0286	1.14 (0.77–1.69)	0.5103
SHS at work **	20.3	9.8	0.42 (0.17–1.04)	0.0610	11.3	13.9	1.26 (0.43–3.71)	0.6788	0.33 (0.08–1.37)	0.1269
Always or sometimes around smokers indoors (home, work, public places)	40.4	41.8	1.03 (0.77–1.38)	0.8367	48.1	45.5	0.93 (0.69–1.25)	0.6246	1.11 (0.73–1.69)	0.6213

^†^ Odds ratio (OR) comparing follow-up to baseline period calculated using Generalized Estimation Equation (GEE); * Significance of the odds ratio of the time period; ^‡^ Relative odds ratio (ROR) is the ratio of the odds ratio for the intervention group to the odds ratio for the control group; **^§^** Significance of the ROR. If the estimation of the ROR is different from one we can conclude that the effect of the intervention is significant; ** The number of women who work outside the house was 147 and 207 in the intervention group (baseline and follow-up period) and 227 and 154 in the control group (baseline and follow-up period). CI: Confidence Interval. SHS: second hand smoking.

**Table 2:** Effect of intervention on secondhand smoke exposure on non-smokers and quitters in Argentina and Uruguay.

	Intervention Group (5 Clusters)				Control Group (5 Clusters)				Intervention Effect	
**Argentina**	**Baseline Rate (*n* = 700)**	**Follow-Up Period Rate** **(*n* = 792)**	**OR ^†^**	*p*-Value *	**Baseline Rate (*n* = 768)**	**Follow-Up Period Rate** **(*n* = 705)**	**OR ^†^**	*p*-Value *	**ROR ^‡^ (95% CI)**	***p*-Value ^§^**
SHS at home	25.7	17.8	0.65 (0.5–0.84)	0.0012	24.7	15.1	0.55 (0.43–0.7)	<0.0001	1.18 (0.82–1.69)	0.3659
SHS at work **	25.3	9.1	0.28 (0.1–0.81)	0.0186	12.9	14.4	1.13 (0.34–3.73)	0.8389	0.25 (0.05–1.23)	0.0881
Always or sometimes around smokers indoor (home, work, public places)	38.7	39.4	0.99 (0.62–1.57)	0.9500	51.6	47.2	0.89 (0.53–1.5)	0.673	1.1 (0.5–2.21)	0.7849
**Uruguay**	**(*n* = 443)**	**(*n* = 475)**	**OR ^†^**	*p*-Value *	**(*n* = 548)**	**(*n* = 580)**	**OR ^†^**	*p*-Value *	**ROR ^‡^ (95% CI)**	***p*-Value ^§^**
SHS at home	19.0	22.5	1.25 (0.93–1.68)	0.1446	21.0	21.6	1.02 (0.77–1.36)	0.8944	1.22 (0.81–1.85)	0.3372
SHS at work	9.1	11.3	1.08 (0.43–2.73)	0.8665	6.1	12.1	2.11 (0.19–23.34)	0.5427	0.51 (0.04–6.74)	0.6117
Always or sometimes around smokers indoor (home, work, public places)	43.1	45.7	1.11 (0.89–1.38)	0.3563	43.1	43.4	0.98 (0.84–1.14)	0.8027	1.13 (0.87–1.47)	0.3678

^†^ OR comparing follow-up to baseline period calculated GEE; * Significance of the odds ratio of the time period; ^‡^ ROR is the ratio of the odds ratio for the intervention group to the odds ratio for the control group; **^§^** Significance of the ROR. If the estimation of the ROR is different from one we can conclude that the effect of the intervention is significant; ** The number of women who work outside the house was 101 and 142 in the intervention group (baseline and follow-up period) and 176 and 120 in the control group (baseline and follow-up period).

**Table 3:** Effect of the intervention on secondhand smoke exposure on attitudes for non-smokers and quitters.

	Intervention Group (10 Clusters)				Control Group (10 Clusters)				Intervention Effect	
	Baseline Rate (*n* = 1143)	Follow-Up Period Rate (*n* = 1267)	OR ^†^	*p*-Value *	Baseline Rate (*n* = 1316)	Follow-Up Period Rate (*n* = 1285)	OR ^†^	*p*-Value *	ROR ^‡^ (95% CI)	*p* Value ^§^
Always or sometimes try to avoid breathing smoke expelled by people smoking near you	86.8	87.4	1.07 (0.89–1.29)	0.4719	87.6	87.4	0.99 (0.7–1.4)	0.9525	1.08 (0.73–1.6)	0.6905
There are rooms where smoking is forbidden	91.5	92.6	1.16 (0.9–1.51)	0.2486	89.5	93.4	1.61 (1.2–2.15)	0.0015	0.72 (0.49–1.07)	0.1062

^†^ ORGEE; * Significance of the odds ratio of the time period; ^‡^ ROR is the ratio of the odds ratio for the intervention group to the odds ratio for the control group; **^§^** Significance of the ROR. An ROR estimate statistically different from one implies a significant intervention effect.
